# DNA strand displacement based computational systems and their applications

**DOI:** 10.3389/fgene.2023.1120791

**Published:** 2023-02-22

**Authors:** Congzhou Chen, Jinda Wen, Zhibin Wen, Sijie Song, Xiaolong Shi

**Affiliations:** ^1^ School of Computer Science, Beijing University of Technology, Beijing, China; ^2^ Institute of Computing Science and Technology, Guangzhou University, Guangzhou, China

**Keywords:** DNA strand displacement, DNA computing, integrated circuits, artificial neural networks, cancer detection

## Abstract

DNA computing has become the focus of computing research due to its excellent parallel processing capability, data storage capacity, and low energy consumption characteristics. DNA computational units can be precisely programmed through the sequence specificity and base pair principle. Then, computational units can be cascaded and integrated to form large DNA computing systems. Among them, DNA strand displacement (DSD) is the simplest but most efficient method for constructing DNA computing systems. The inputs and outputs of DSD are signal strands that can be transferred to the next unit. DSD has been used to construct logic gates, integrated circuits, artificial neural networks, etc. This review introduced the recent development of DSD-based computational systems and their applications. Some DSD-related tools and issues are also discussed.

## 1 Introduction

DNA, as a natural material, is biocompatible and programmable. With the development of biotechnology, DNA can be precisely synthesized, controlled, and detected by various tools (Beckwitt et al., 2018; Palluk et al., 2018; Del Grosso et al., 2020). Its sequence can be programmed as a 4-bit of encoding information (ATGC four nucleotides), which is information denser than the 2-bit electronic devices (0 and 1) (Ceze et al., 2019). In this strategy, 1 gram of DNA can store about one EB of data (Sun et al., 2019). Furthermore, DNA, as an information storage carrier, can be kept at −25°C for 10,000 years. These properties make DNA a perfect information material.

DNA can be used to construct many computational devices. Their sizes are predictable and controllable. The length of one double helix of B-type DNA is 3.4 nm, and the width is 2 nm. A single strand of DNA is flexible, whereas the double strand of DNA is among the stiffest polymers, with a persistence length of 50 nm in 0.1 M aqueous NaCl (Chirkov et al., 2022). Therefore, DNA strands and their complexes can be utilized as computational devices (Chen et al., 2020; Chen et al., 2022a; Xu et al., 2022).

So far, many DNA computing models have been proposed. According to DNA structures, these models can be classified as the single-strand-based computational model (Adleman, 1994; Liu et al., 2000), DNA-tile-based model (Mao et al., 2000; Rothemund et al., 2004), DNA origami-based model (Woods et al., 2019; Amir et al., 2014), and mixture model (Xu, 2016). Among them, the single-strand-based computational model is the simplest and easiest method to construct. Researchers do not have to design complex or large structures. Single DNA strands are utilized as the inputs and outputs, which can be programmed and cascaded to solve complex problems. The most popular single-strand-based computational method is DNA strand displacement (DSD) reaction.

**FIGURE 1:**
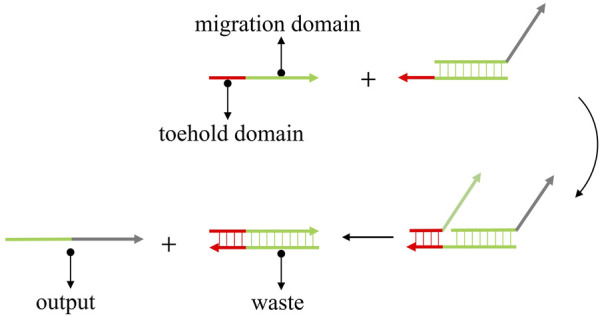
The mechanism of toehold-mediated strand displacement. The input strand reacts with DNA complex and produces the output strand.

DSD is the ideal technology for the single-strand-based computational model. DSD was first proposed by Yurke et al. (2000). They constructed a DNA tweezer that can transfer two states *via* DNA strand displacement reactions. DSD involves a short single-strand domain (toehold domain) and the replacement of paired double strand (migration domain). The input strand can react with the DNA complex that is mediated by the toehold domain and produce the output strand. As shown in Figure 1. The red part is the toehold domain; the green part is the migration domain. The input strand consists of a single strand with the toehold and an unpaired migration domain. While the DNA complex contains an unpaired toehold domain and a paired migration domain. The input strand will react with the complex, beginning in the toehold domain. The mechanism of DSD involves the strand’s thermodynamic stabilization process. The incompletely paired strands (DNA complex) will be replaced by fully paired strands.

DSD reactions can be implemented to solve computational problems. Many mathematical operations, such as exponential operations (Salehi et al., 2018), multiplication operations (Genot et al., 2013), Boolean operations (Zhao et al., 2021), and satisfiability problems (Liu et al., 2000) have been completed *via* DSD. Additionally, DSD reactions are the approximate representation of arbitrary chemical reactions. As a result, many DSD-based chemical reaction systems have been built, including oscillators, chaotic systems, and feedback digital logic (Soloveichik et al., 2010). DSD reaction is enzyme-free, and it can be used for logical gates, including AND, OR, YES, NOT, NOR, NAND, XOR, Threshold, Inhibited gates, etc. (Okamoto et al., 2004; Seelig et al., 2006; Carell, 2011; Song et al., 2019). The DSD-based gates can be cascaded into integrated circuits to solve complex computing problems. What’s more, integrated DSD circuits can function as artificial neural networks (ANN) and perform machine learning (ML) algorithms. The Multilayer Perceptron (MLP) (Arredondo and Lakin, 2022), Support Vector Machine (SVM) (Lopez et al., 2018), Hopfield network (Qian et al., 2011), and Convolutional Neural Network (CNN) (Xiong et al., 2022) had been architected *via* DSD reactions.

DSD can be combined with other technologies and utilized in a wide range of applications. So far, DSD has been combined with CRISPR technology (Ishino et al., 1987; Montagud-Martínez et al., 2021), DNA origami (Rothemund, 2006; Zhang et al., 2022), enzymes (Bucci et al., 2022; Schaffter and Strychalski, 2022), proteins (Fern and Schulman, 2017), etc. These combinations effectively expand the application scenarios for DSD. It has been applied in information storage (Lin et al., 2020; Banal et al., 2021), encryption (Zhu et al., 2022), medical treatment (Peng et al., 2018), biosensing (Li et al., 2021), etc.

**FIGURE 2:**
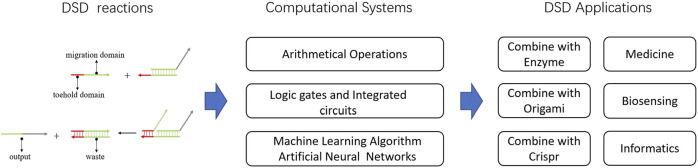
Abstract of DNA strand displacement System. The DSD-based computational systems and their applications were introduced.

In this review, we first introduced the DSD computing systems and their ability to solve computational issues. Focusing on DSD integrated circuits, DSD-based artificial neural networks. Then, we presented the applications of DSD, including molecules and technologies that combine with DSD. Last, we introduced some useful tools for DSD. Defects and problems of DSD were also discussed. Figure 2 shows the abstract of this review.

## 2 DSD computational systems

### 2.1 Arithmetical operations

Fractional coding and Matrix multiplication are two important operations for the computing system. Fractional coding is the foundation of other complex arithmetical operations. It is a numeric format for representing numbers with a decimal part. While matrix multiplication is vital for the implementation of deep networks. Matrix operations are widely used in deep learning because they are an efficient way to represent and manipulate complex mathematical relationships.

Salehi et al. (2018) constructed two types of fractional coding. One is the unipolar fractional coding, the other is the bipolar fractional coding. The definition is illustrated as formulas 1, 2:

$$x=\frac{\left[X_{1}\right]}{\left[X_{0}\right]+\left[X_{1}\right]}$$ (1)

x is the variable. A pair of molecular $X_{0}$ and $X_{1}$ is assigned to x . The value of the variable is determined by the ratio of the concentration for the assigned pair. Therefore, the value of x is confined to unit interval [0, 1]. This strategy is unipolar fractional coding.

$$x=\frac{\left.\left[X_{1}\right]-{\left[X\right.}_{0}\right]}{\left[X_{0}\right]+\left[X_{1}\right]}$$ (2)

**FIGURE 3:**
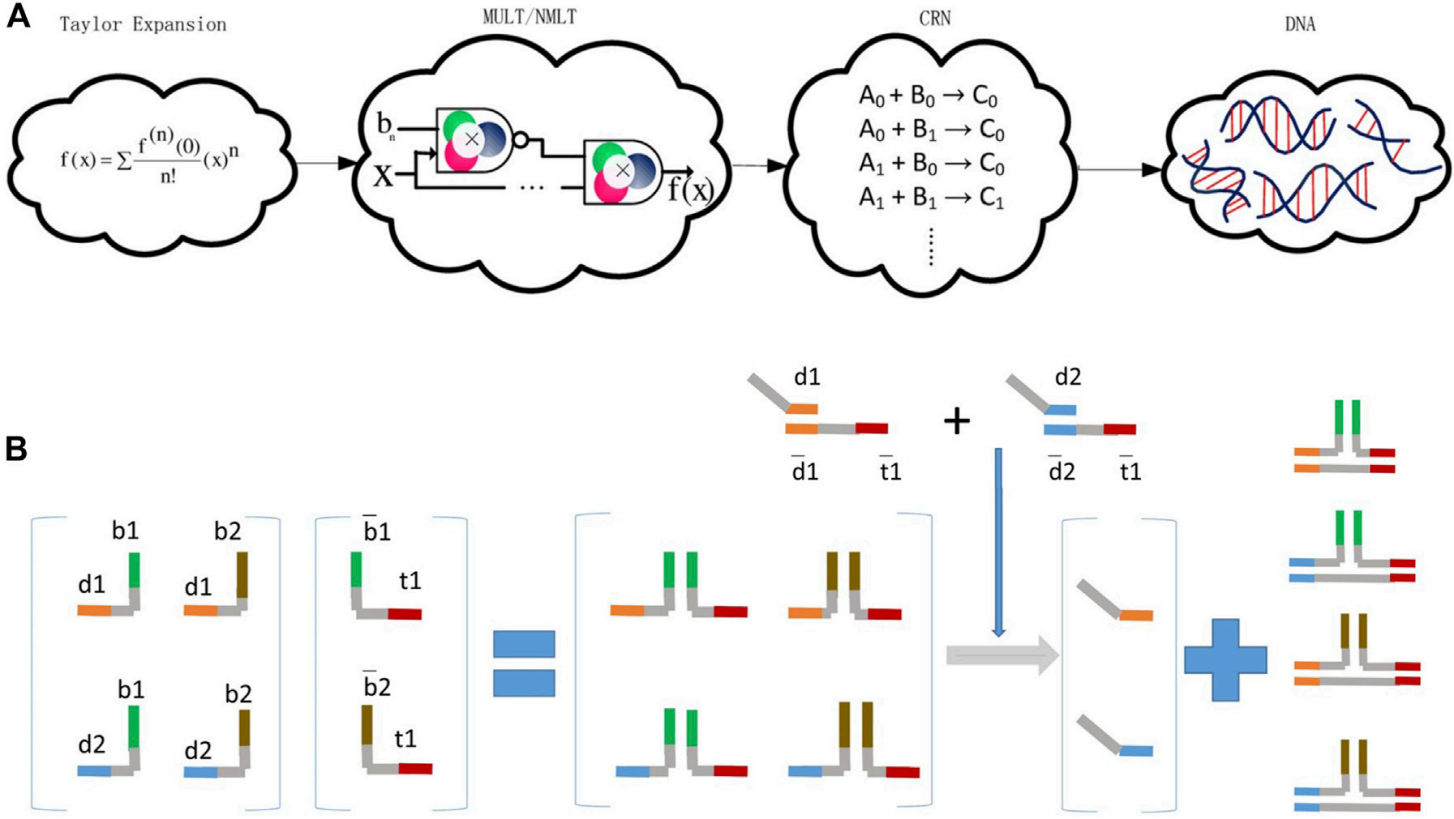
Arithmetical operations are executed by DSD reactions. **(A)** Mathematical functions convert to Taylor expansion and are executed by DSD chemical reaction network (CRN). Reproduced with permission (Palluk et al., 2018). Copyright 2018, Nature Publishing Group. **(B)** Matrix multiplication operated by DSD. The multiple of matrix operation is calculated by strands reactions. Reproduced with permission (Genot et al., 2013). Copyright 2013, John Wiley and Sons Ltd.

Similarly, the value of the variable x locates in the range of [−1, 1], represented by formula 2. This is the definition of bipolar fractional encoding. Further, they implemented mathematical functions, including exponentials, sigmoid, sine, cosine, and tanh functions (Salehi et al., 2018). They defined the five basic DSD reactions as the multiplication (Mult), Nor-Mult (NMult), multiplexer, bipolar Mult, and bipolar NMult units. Then, mathematical functions can be transformed into Taylor expansions, and constructed by these five units, Figure 3A.

Matrix multiplication can be performed *via* DSD reactions. Reference (Genot et al., 2013) is the first research that explicitly illustrated the implementation of matrix multiplication with DSD. Genot et al. (2013) designed the typical multiplication of a 2 × 2 vector by a 2 × 1 matrix. They utilized the combinations of toehold and migration domains, and implemented the multiplication of a 2 × 2 vector by a 2 × 1 matrix. In this research, toehold and migration domains were dynamically and combinatorially linked to form DNA complexes, which represent the matrices. n toehold domains and n substitution domains can form $n^{2}$ substitution complexes, which greatly reduce the number of required chains and increase the computational power. The operation processes are shown in Figure 3B, the multiplication

$$\left[\begin{array}{cc} M_{11} & M_{12}\\ M_{21} & M_{22} \end{array}\right]\times \left[\begin{array}{c} X_{1}\\ X_{2} \end{array}\right]=\left[\begin{array}{c} Y_{1}\\ Y_{2} \end{array}\right]$$ (3)

The elements in Metrix M were represented by strands { $d_{1}b_{1},d_{1}b_{2},$ $d_{2}b_{1},$ $d_{2}b_{2}$ }, Metrix X was represented by { ${\bar{b}}_{1}t_{1},$ ${\bar{b}}_{2}t_{2}$ }, the cap means its complementary sequences. The output of this matrix multiplication is matrix Y, represented by two strands { $Y_{1},Y_{2}$ }.

DSD-based chemical reaction networks can be designed in a programmable language for solving mathematical problems. Tang et al. (2021) designed weighted reactions, sum reactions, threshold modules *via* DSD, and solved a three-parameter 0–1 knapsack problem. Lopiccolo et al. (2021) implemented a last-in, first-out stack structure *via* DSD. This stacked structure stores two signals, and signals are released into the solution by order of the input strand. Later, the stack can be rebooted by the activation strand.

### 2.2 Logic gates and integrated circuits

**FIGURE 4:**
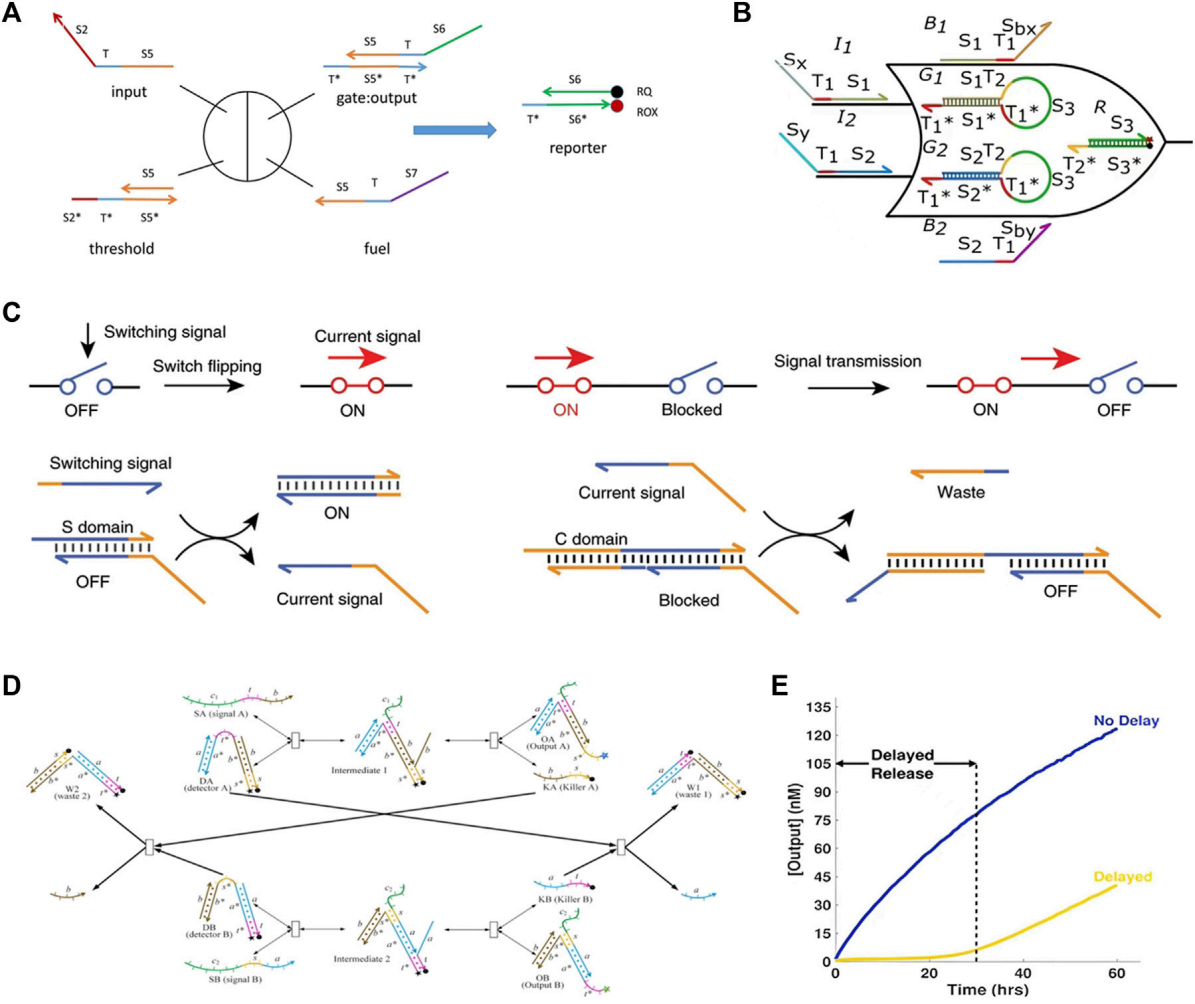
DSD logic gates and integrated circuits. **(A)** The seesaw gate. It consists of the input, threshold, output gate and report Reproduced with permission (Qian and Winfree, 2011). Copyright 2011, The American Association for the Advancement of Science. **(B)** The 2-input renewable circuit. The hairpin gate in this circuit is renewable. Reproduced with permission (Eshra et al., 2019). Copyright 2019, IEEE Publishing Group. **(C)** DNA switching circuits (DSCs), DSD gate switched its state according to the input signal. Reproduced with permission (Wang et al., 2020). Copyright 2020, Nature Publishing Group. **(D)** The cross-inhibit gate. Signals A and B are crosses inhibited. Reproduced with permission (Liu et al., 2020). Copyright 2020, Oxford University Press. **(E)** Time-delayed circuits. Target strand release into the solution for days. Reproduced with permission (Fern et al., 2017). Copyright 2017, American Chemical Society.

Logic gates are the primary unit of integrated circuits. They are the foundation of modern computer systems. DSD and modified DSD reactions can be programmed and utilized as logic gates. An outstanding research of improved DSD reaction is the seesaw gate, proposed by Qian and Winfree (2011). The mechanism of the seesaw gate is the reversible DSD reaction. Figure 4A depicts the reaction principle. The input strand will first react with a threshold gate until all the thresholds are consumed. As the threshold chain has a longer toehold domain, which guarantees the first step. Then, excessive input can react with the output gate and produce the output strand. Last, the output strand will react with the report gate and give the fluorescent report signal. Because the fuel strand and input strand have the same toehold and migration domain. Fuel strand can replace the input strand out from the byproduct of second step.

The renewable gThere are many topics involving DSD logic gates, including callability, signal restoration, time-responsive, etc. ate is one of them. Eshra et al. (2017) modified the seesaw gate motif into a hairpin, called the “DNA hairpin-seesaw gate”. As shown in Figure 4B, the inner two hairpin motifs. The sequences of them from 5′ to 3′ are {S_1_, T_2_, S_3_, T_1_^*^, S_1_^*^, T_1_^*^} and {S_2_, T_2_, S_3_, T_1_^*^, S_2_^*^, T_1_^*^} (cap “*”represents the complementary sequences). S_1_ pairs with S_1_^*^ to form the hairpin, S_2_ pairs with S_2_^*^ to form the other hairpin. The two toehold domains T_1_^*^ and T_2_ locate at the two sides of these motifs, similar to the original seesaw gate. Further, they added two extractors to initialize the hairpin-seesaw gate, which realized the renewable process. This hairpin gate can be reused more than three times in consecutive calculations. They constructed a 2-input renewable circuit *via* this motif (Eshra et al., 2019), as shown in Figure 4B. Based on DSD’s AND gate and XOR gate, half-adder or full-adder circuits can be assembled. Xiao et al. (2020) constructed a three bits full-adder . Xie et al. (2022) used three dual-track logic gates and assembled a four bits full-adder.

In 2020, Wang et al. (2020) designed DNA switching circuits (DSCs). The input chain interacts with the gate to generate an output strand. Then the output strand will propagate and arrive at the next gate. Gates will change their states according to the output strand. Therefore, the ON and OFF states are switched, which can represent Yes and No. The output strand propagates like a current passing through the DSCs’ gate. Besides, the DSC scheme does not use the dual-track strategy to design NOT gates. (Dual-track strategy refers to a design methodology used in digital circuits that employs two parallel design approaches or paths. The two paths operate in parallel and regularly interact with each other so that both are progressing toward the final goal. For example, the two paths of full-adder are add-path and carry-path. Furthermore, the full-adder is the fundamental component of digital computing.) As a result, its required chains are reduced by 3/4 compared to current DSD circuits. The implementation scheme is shown in Figure 4C.

Liu et al. (2020) created a cross-inhibit gate and then used it to perform four-input time-sensitive circuits. Signal strand A can react with detector DA to produce output OA and kill strand KA, as shown in Figure 4D. Afterward, KA can react with detector DB, which inhibits the reaction of DB and signal strand B. As a result, signal A inhibited signal B. If signal B is first added, then the situation is the opposite. This DSD-based cross-inhabit strategy is a simple and effective method for constructing time-sensitive circuits. Fern et al. (2017) also designed time-delayed DSD circuits. They created a simple DSD circuit with eight strands that can release target DNA strands into solution at a constant rate for hours to days. The result is illustrated in Figure 4E.

### 2.3 ML and ANN algorithms

**FIGURE 5:**
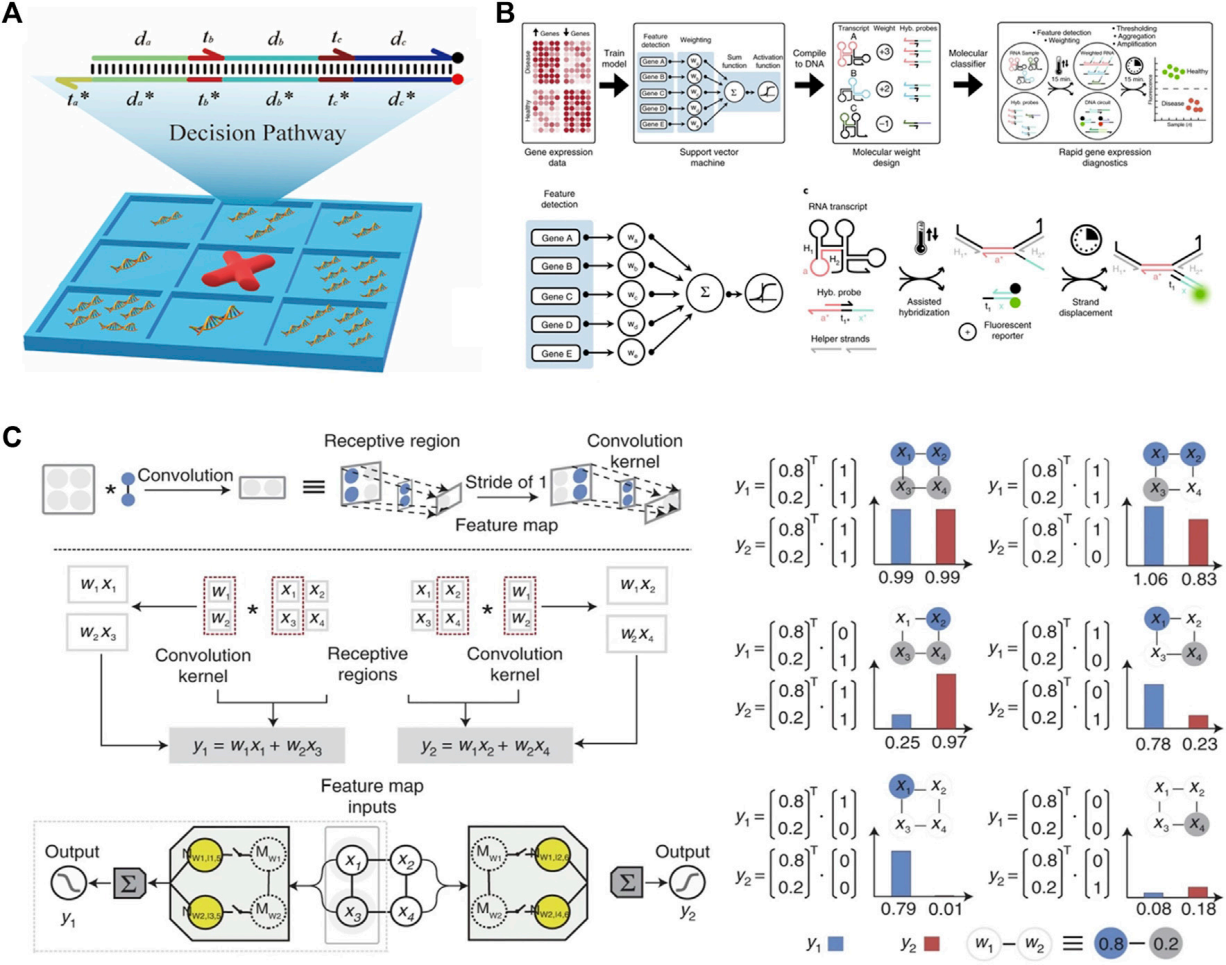
Machine learning and ANN algorithm constructed *via* DSD. **(A)** The tic-tac-toe game was implemented *via* DSD. This DSD complex represents a four-steps one-result decision pathway. Reproduced with permission (Chen et al., 2022b). Copyright 2022, American Chemical Society. **(B)** The SVM algorithm was performed *via* DSD. The five selected genes transcript to their RNA and react with the DSD complex. Reproduced with permission (Lopez et al., 2018). Copyright 2018, Nature Publishing Group. **(C)** The CNN system was implemented *via* DSD. The left part illustrates the Metrix multiplication process. The right part shows the convolution results. Reproduced with permission (Xiong et al., 2022). Copyright 2022, Nature Publishing Group.

Machine learning algorithms and artificial neural networks can be implemented *via* DSD reactions. The decision tree is a classical classification ML algorithm. It processes the classification pathway based on the known probability of occurrence situations. Its classification pathway can be executed *via* DSD reactions. Chen et al. (2022b) designed a domino-like DSD sequential system, which can execute four steps of the decision pathway. As shown in Figure 5A.

Support Vector Machine (SVM) is another powerful ML algorithm. It is a two-class classification algorithm. Its basic approach is to find the maximum classification interval in the feature space. Lopez et al. (2018) performed a gene classification SVM system. The selected genes for each class are pretrained on a silicon computer. Then, these genes transcript to RNA and reaction with DNA complexes under the guidance of SVM algorithm. The weights of each gene are implemented by transcript times, as shown in Figure 5B. Using seesaw gates and the dual-track strategy, Qian et al. 2011 further constructed a Hopfield network. This network contains twenty-four circuits and “remembers” four patterns.

In 2018, Qian et al. extended the computational border of the seesaw gate. They constructed a winner-take-all neural network without using the dual-track strategy (Cherry and Qian, 2018). This network is a three-layer, fully connected artificial network. The inputs are 100 bits DNA strands in 10×10 patterns. The hidden layer consists of 20 distinct DNA molecules that can react with these 100 bits DNA strands *via* DSD reactions. The output layer shows the classification results of handwritten digits ‘1’ to ‘9’. This is the first research attempt using DSD to construct complex ANN.

Convolutional Neural Network (CNN) can also be implemented *via* DSD reactions. The essence of the convolution operation is matrix multiplication. Xiong et al. (2022) take the same matrix multiplication strategy as Genot et al. (2013). They designed the Metrix operation DSD system, including the multiplication of two matrices, matrix Addition, and matrix Subtraction.

The matrix multiplication is $\left|X_{2\times 2}\right|\times \left|W_{2\times 1}\right|$ , results are $y_{1}$ and $y_{2}$ . Then, $y_{1}$ and $y_{2}$ are added or subtracted to produce the final results Y which is the result of this convolution process. They implemented this kernel $\left|W_{2\times 1}\right|$ to convolute the input ‘image’ (image is transferred to 144 bits DNA strands in 12×12 patterns). Then the image indentation result can be illustrated by $\overset{n}{\underset{1}{\sum }}Y$ (n is the convolutional times). As shown in Figure 5C. Using this DSD-based CNN method, they successfully identified oracle bones as well as English letters and Arabic numerals.

## 3 Applications of DSD computing

DNA is biocompatible, and DNA structures can be endocytosed by cells. Furthermore, DNA can be modified and linked with drugs, proteins, and other molecules. DSD computational systems combined with molecules and biotechnologies have applications in medicine, biosensors, informatics, and other fields.

### 3.1 DSD combines with CRISPR technology

CRISPR (Clustered Regularly Interspaced Short Palindromic Repeats) was first found from *Escherichia coli* bacteria by Ishino et al. (1987). The CRISPR system consists of an artificially designed sgRNA (single-guide RNA) and a Cas protein, which combine to form a complex. sgRNA has a guide sequence of about 20 nt that matches the target gene, then the PAM sequence in the upstream can cleaved, repressed, and activate the target gene with the help of Cas protein. As sgRNA is a segment of RNA around 100 nt, it is possible to program it.

The combination of CRISPR and DSD enables some in-cell logical circuits, even intracellular gene regulation can be realized.Jin et al. (2019) added the toehold domain for sgRNA at the 5′ end (guide sequence) and 3′ end (scaffold structure) without affecting its activity. Because the guide sequence of sgRNA is sensitive to its formation, the binding and unbinding of the toehold domain can be utilized for controlling its activity. Then, they used this designed CRISPR as the switch to control a DSD system.

**FIGURE 6:**
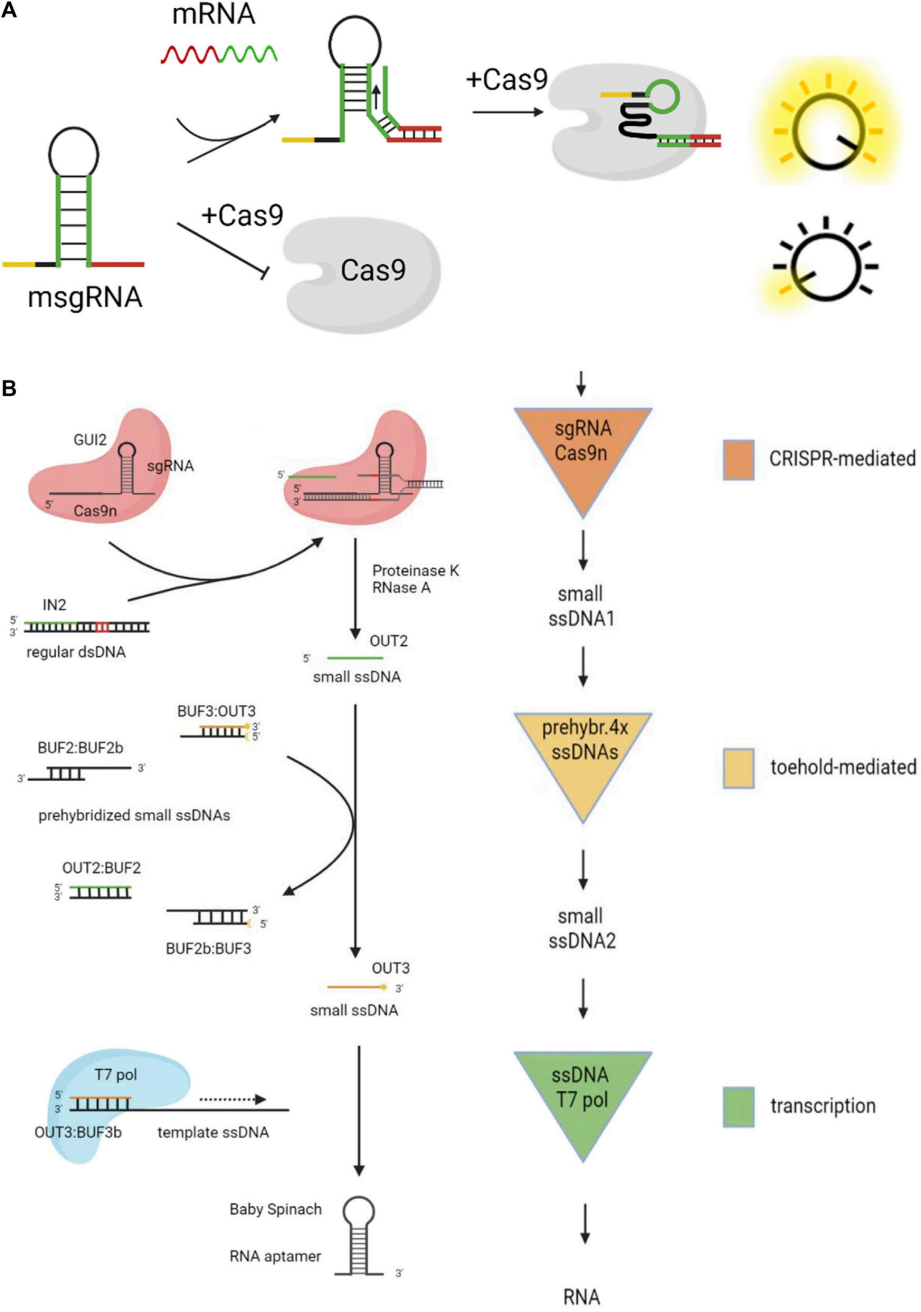
DSD combinates with CRISPR technology. **(A)** The structure of sgRNA was designed for DSD system.Reproduced with permission (Li et al., 2019). Copyright 20,219, American Chemical Society. **(B)** The CRISPR Cas9 system was used to cut the byproduct (dsDNA) of DSD reaction. Reproduced with permission (Montagud-Martínez et al., 2021). Copyright 20,219, American Chemical Society.

Li et al. (2019) designed the scaffold structure of sgRNA, as shown in Figure 6A. Regular sgRNA consists of a guide sequence (the yellow part) and a scaffold sequence (the black part). They added the mRNA sensing sequence (the green part) and the toehold domain (the red part) in the sgRNA, which is named msgRNA. This structure was designed to disrupt the scaffold of sgRNA, making it impossible to bind with the Cas9 protein. When mRNA is added, the strand displacement reaction opens the hairpin of msgRNA, restoring sgRNA activity. Hao et al. disrupted the structure of sgRNA through a blocking strand. Then they added a replacement strand to react with the blocking strand, thus restoring the activity of sgRNA (Hao et al., 2020).

The fully paired double-stranded DNA (dsDNA) is generally considered the waste product of the DSD reaction. However, Roser et al. made the waste strands useful through CRISPR technology (Montagud-Martínez et al., 2021), as shown in Figure 6B. The regular dsDNA is cut by CRISPR Cas9 and produces output2. Then output2 will react with prehybridized ssDNAs through DSD reaction and produce output3. Last, output3 can regulate the target gene with the help of T7 pol. The whole system includes CRISPR-mediated reactions, toehold-mediated reactions, and the transcript process.

From the above-mentioned designs, we can conclude that DSD reactions can be used as the switch to control the CRISPR process. These above-mentioned studies have a common strategy, which is to program the DSD reaction domain in sgRNA sequences. This strategy is direct and efficient.

### 3.2 DSD combines with molecules

Based on DSD reactions, DNA structures can be designed as molecular robots, molecular machines, and many other molecular devices. These devices can be precisely controlled *via* the DSD reaction. DSD combines with various molecules can make these devices efficient for many applications.

#### 3.2.1 DSD combines with origami

DNA origami is a powerful tool for designing arbitrary shapes of DNA structures (Rothemund, 2006). It involves a long scaffold strand and hundreds of short strands that help to bind to predesigned structures. Every location of the origami structure can be programmed through the binding strands. Therefore, DNA origami can be utilized as the platform for DSD reactions.

**FIGURE 7:**
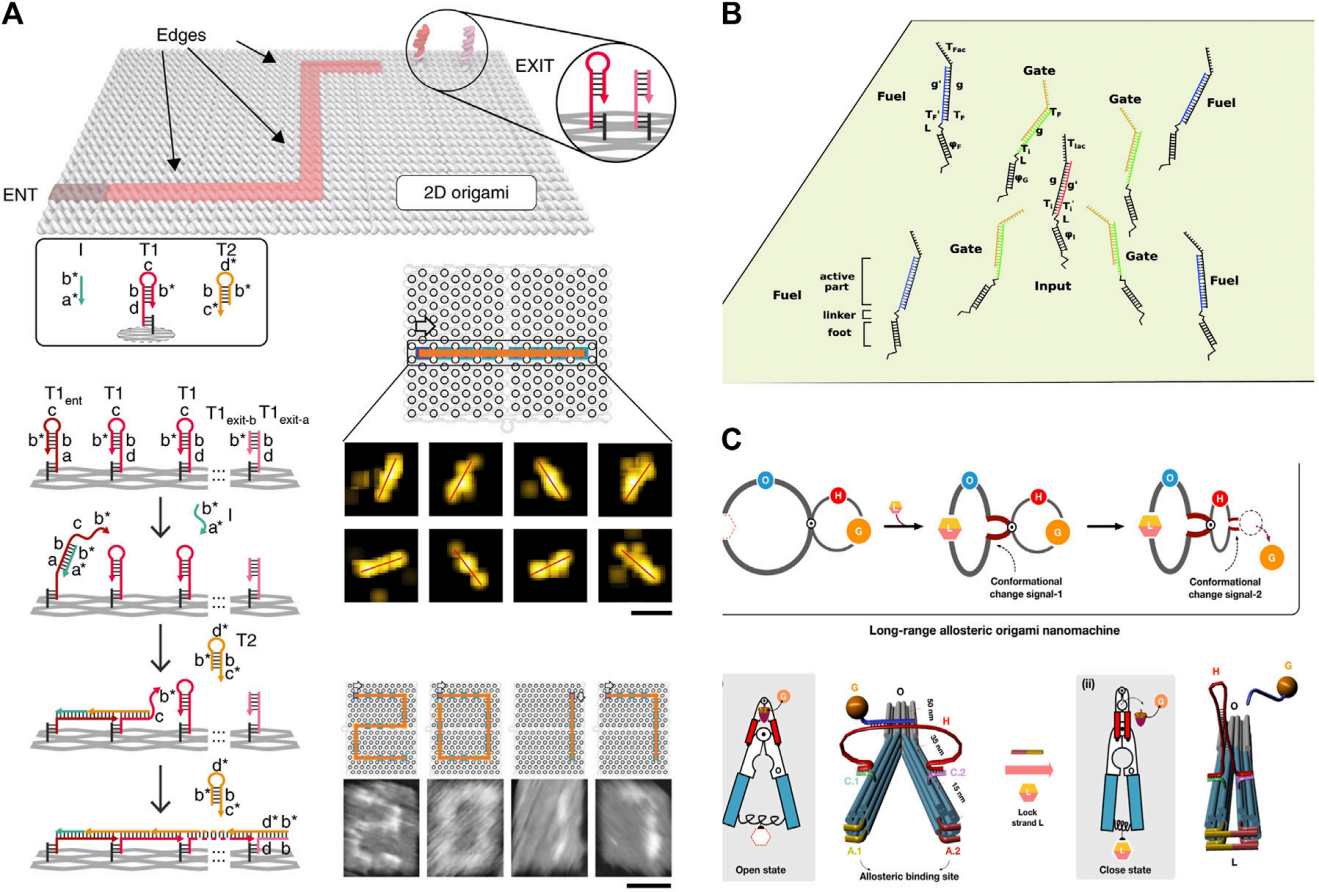
DSD combinates with DNA origami structures. **(A)** The DNA cruising robot walks on the origami platform through DSD reactions. Reproduced with permission (Chao et al., 2019). Copyright 2019, Nature Publishing Group. **(B)** The DSD logic gates that implemented on the origami platform. Reproduced with permission (Ruiz et al., 2015). Copyright 20,219, Royal Society of Chemistry. **(C)** DSD as the switches to control a long-range allosteric origami nanomachine. Reproduced with permission (Zhang et al., 2022). Copyright 2022, Nature Publishing Group.

Lund et al. (2010) designed a molecular robot; called the molecular spider. The three legs of this spider are specific DNA enzyme strands that can cruise on a DNA origami *via* DSD reactions. Chao et al. (2019) designed a DNA cruising robot that enables molecular reactions along the designed paths on a DNA origami platform. The robot can find the paths of the DNA origami maze by the controlled DSD reactions. They observed all the walking paths of the DNA cruising robot in the maze using AFM and DNA-PAINT imaging characterization. This DSD and origami combination method provides a strategy for single-molecule diagnosis and treatment. As shown in Figure 7A. Ruiz et al. (2015) tethered DSD system on a DNA origami platform, making the reactions faster than reactions in solution. Besides, reactions between gates are limited among their neighbors. They then devised many logic gates through this method. As shown in Figure 7B.

Zhang et al. (2022) used the DSD reactions as switches to control an origami nanomachine. They designed a long-range allosteric origami nanomachine. DNA complexes were programmed at the binding sites. Then the allosteric nanomachine can open or close by the input strands *via* DSD reactions. As shown in Figure 7C.

DSD reaction is hard to visualize. The common method uses the fluorescent signal as the reporter. However, the above-mentioned devices made these processes visual. The DSD switch on/off status can be represented by the shapes of origami. The track paths of the cruising robot and the spider on the DNA origami substrate can be visualized *via* AFM. When DSD combines with origami, these devices become interesting and credible.

#### 3.2.2 DSD combines with enzymes

Enzymes in DSD computational systems can be used as regulators to control their reactions. Bucci et al. (2022) designed a blocker strand that can bind to the toehold domain, which prevented the DSD reactions. Then they took two steps to recover the activity of DSD. First, they used the RNase H enzyme to cut the block strand, thus exposing the toehold domain. Second, they used formamidopyrimidine DNA glycosylase or uracil-DNA glycosylase to degrade the block strand. The degradation rates of these two glycosylases are different. Therefore, the reaction rate of the DSD system can be controlled.

With the help of transcriptase, DSD system can be deployed in cells. Schaffter et al. (Schaffter and Strychalski, 2022) designed a DNA strand with a specific sequence that can be transcript to RNA complexes in cells. This specific DNA strand is endocytosed by cells, and then transcripted in cells. Therefore, the entire RNA circuits was implemented in cells. Fern et al. (Fern and Schulman, 2017) designed a hairpin structure at the 5′ and 3′ end of the DSD complexes, which can prevent the binding enzymes from being disrupted by other proteins in serums.

Enzymes can regulate the expression or reaction of the DSD system. With the help of enzymes, a sophisticated regulatory network with multiple coordinated functions can be realized. However, the redundant enzyme could complicate the reaction environment, which might cause instability in the system.

### 3.3 DSD application in informatics

The ATGC four nucleotides can be used as 4-bit coding information, and synthetic DNA strands have been utilized as data storage material. Generally, a strip of DNA data consists of an addressing domain, an information domain, and a correction domain. DSD reactions can be deployed on these domains. The input strand of the DSD can be utilized as the information encryption, information reader, initiator, etc. The DSD-based information processing is simple and effective.

**FIGURE 8:**
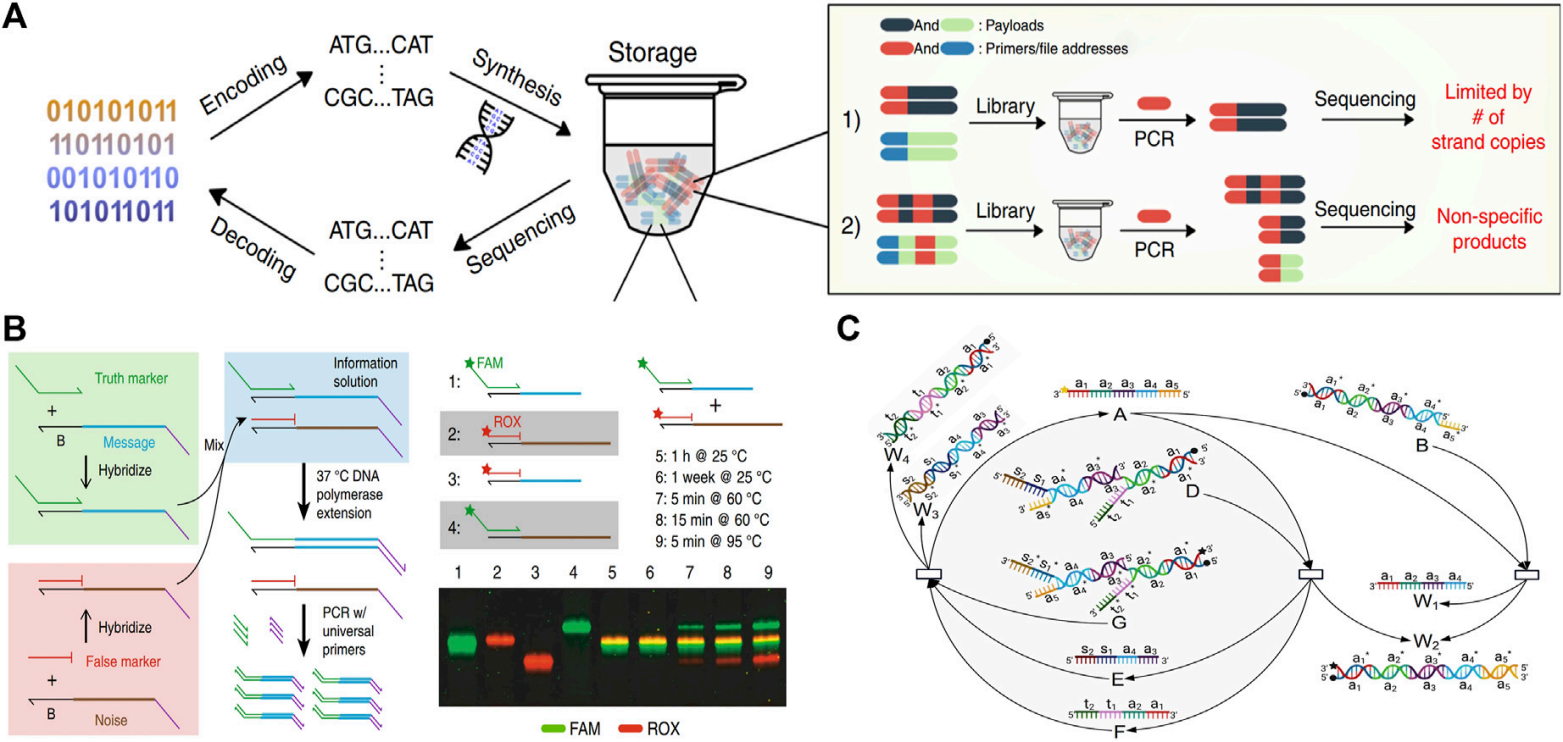
DSD applications in data storage and encryption. **(A)** T7 promoter and a single overhang strand to perform the DNA information storage process. Reproduced with permission (Lin et al., 2020). Copyright 2020, Nature Publishing Group. **(B)** Using DSD reactions as the data deletion process. Reproduced with permission (Kim et al., 2020). Copyright 2020, Nature Publishing Group. **(C)** Key of DNA information is transferred through the DSD degradation reaction. Reproduced with permission (Zhu et al., 2022). Copyright 2022, MDPI Publishing Group.

Lin et al. (2020) inserted a T7 promoter and a single overhang strand into the information domain. The overhanging strand can accomplish the tagging, locking, replacement, and deletion processes for the DNA information with the help of the T7 promoter. Additionally, the DNA data can be read out through the PCR process without disrupting the original DNA strand. Lin increases the information capacity and reduces coding complexity by using the DSD system, which avoids the impact of primers on DNA coding regions. As illustrated in Figure 8A.

Kim et al. (2020) used the DSD reactions to extract image data that was stored in DNA strands. They encoded a false message-encoded strand that has the same primers and length as the true message-encoded strand. Correct information can be extracted from the true strands through DSD reactions and the PCR process, while the false strand cannot. However, if the whole system is heated to above 95° and kept for 5 min, the correct information and the error information will be mixed together, resulting in decoding failure, and the related data cannot be recovered, which plays the role of fast erasure of the target information. As shown in Figure 8B.

Banal et al. (2021) sealed DNA information in impervious silica capsules. The surface of silica gel is labeled with single overhanging DNA strands, which represent the characteristics of the stored information. When information retrieval is required, a complementary strand with magnetic bead-modified DNA strands will be added to the solution. These two strands will react through the DSD reaction, and the silica capsule data will be captured by magnetic adsorption.

Zhu et al. (2022) used the DSD reactions as the encryption approach. This encryption approach includes the conversion of information to DNA sequences through Huffman coding. Then, the key to this DNA information is transferred through a degradation reaction. Finally, the DSD transferred key is extended *via* a catalysis reaction, which increases the decryption complexity. The key transfer reactions are illustrated in Figure 8C.

### 3.4 DSD applications in Medicine and Biosensing

DSD can be designed as a tool for cancer detection. DNA strands modified with aptamers can target the cancer cell surface, and the detection signals or drugs can be released through the DSD reaction. In these applications, the ligands target specific cells, and the DSD system is utilized as the switch to turn on the drug release process or cancer detection process.

**FIGURE 9:**
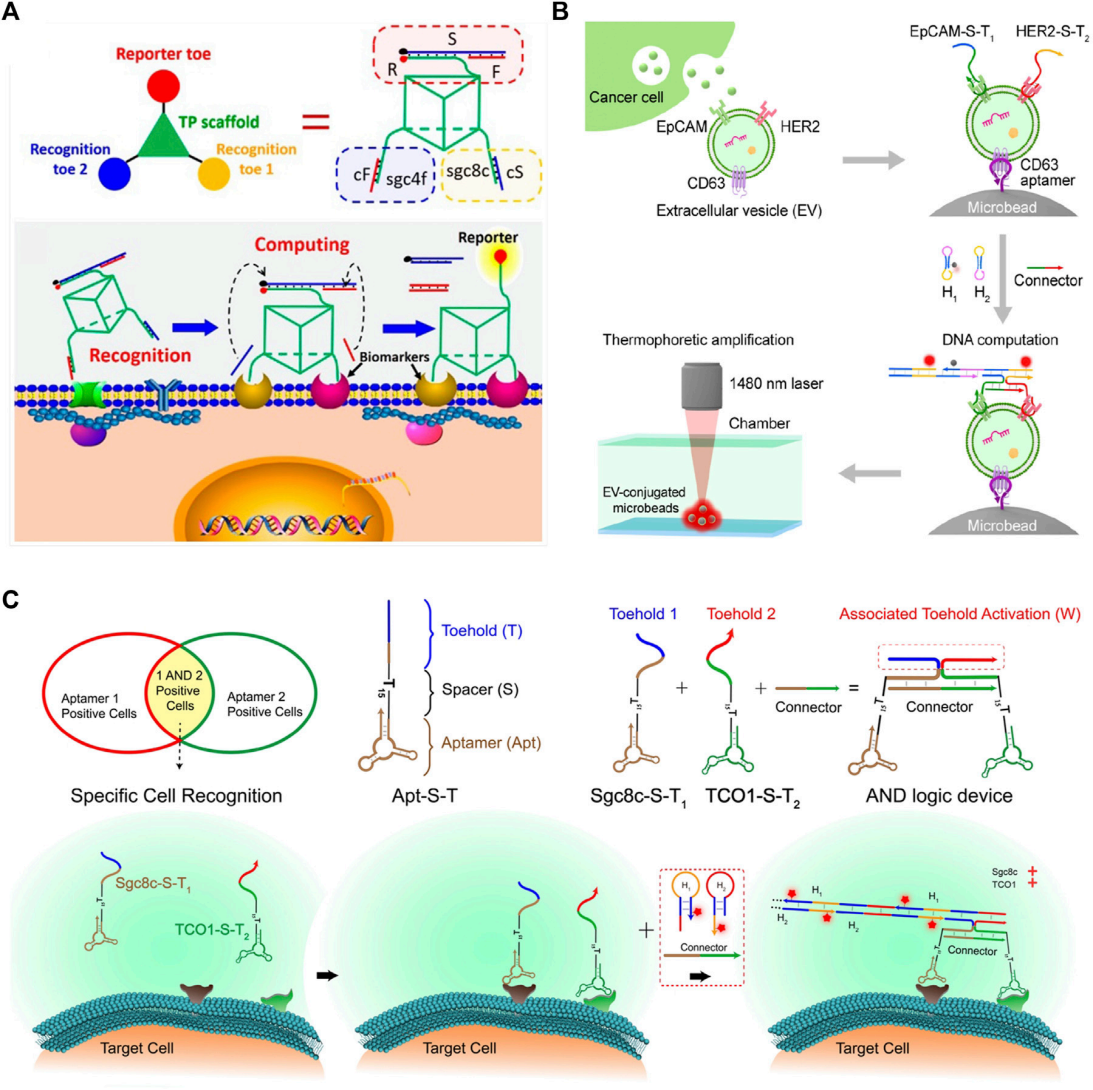
DSD applications in Medicine and Biosensing. **(A)** The 3D DNA nanomachine is attached on the cancer cell surface and detects SELEX cancer cells. Reproduced with permission (Peng et al., 2018). Copyright 2018, American Chemical Society. **(B)** The DNA computational device that can identify tEVs through DSD reactions. Reproduced with permission (Li et al., 2021). Copyright 2021, American Chemical Society. **(C)** The DSD AND gate are attached to the cancer cells. Reproduced with permission (Chang et al., 2019). Copyright 2019, American Chemical Society.

Peng et al. (2018) constructed a three-dimensional DNA-based nanomachine that can target cancer cells. This nanomachine can identify the DNA aptamers that are produced by SELEX cancer cells. When the nanomachine binds to the surface of cancer cells, it will produce the fluorescent signal through the DSD reaction. As shown in Figure 9A. This DSD-based cancer detection method is ultra-sensitive to cancer cells and can be used as a tool for early cancer identification.

Molecular profiling of tumor-derived extracellular vesicles (tEVs) is a vital cancer biomarker. Li et al. (2021) used the thermophoresis mediated DNA computing device to identify tEVS. They constructed an AND gate that consists of EpCAM-S-T2 and HER2-S-T2 proteins. tEVS causes the overexpression of two proteins. The whole tEVSs are binding to a Microbead *via* CD63 aptamer. Last, they added the DNA hairpin strands into the solution, and then the AND gate produced the fluorescent signals, which can represent the expression level of tEVs.

Chang et al. (2019) identified and narrowed down a cancer cell-type subpopulation from large populations of similar cells through the DSD logic device. They programmed two cancer-expression proteins, aptamers Sgc8c-S-T1 and TCO1-S-T2, on an AND logic gate. These two aptamers can identify the target cancer cell and attach themselves to its surface. Then, input strands can react with the AND gate and produce fluorescent signals. The scheme of this method is illustrated in Figure 9B. These two methods (Li et al., 2021)- (Chang et al., 2019) have a similarity. They both used two aptamers to identify cancer and then assembled an AND gate. The final signal reports were accomplished *via* DSD AND gate reactions.

## 4 Tools of DSD

Visual DSD (Lakin et al., 2011) is computer simulation software created for DSD researchers. The authors designed specific syntax conventions for DSD language, which can program DSD complexes or single DNA strands in various situations. The DSD syntax can be programmed and constructed to form complex chemical reaction networks (CRN). Besides, the initial condition, CRN conditions, and other parameters can be set at will. Additionally, there are many thermodynamical algorithms provided for different experiments, such as deterministic simulation, stochastic simulation, spatial simulation, etc. Visual DSD generates all possible reactions and products automatically. It helps researchers construct complex DNA reaction networks without manual design, and the results can be visualized.

Leakages could be occurred among with DSD reactions. If partially paired sequences exist between two DNA complexes, they could react with each other without the help of toehold mediation. Visual DSD does not take the leakage into consideration. Zarubiieva et al. (2022) proposed a leak analysis method for Visual DSD. This method, named DSD leaks, consists of a leak reaction enumeration algorithm and leak probability prediction. They extended the logic programming functionality of Visual DSD.

Badelt et al. (2020) proposed a domain-level DSD reaction simulation software; they named it “peppercorn”. Peppercorn is more general than Visual DSD. It considers the natural connection to nucleic acid biophysics, and it is still suitable for structure analysis. The authors implemented three different algorithms for different situations. They presented an enumeration algorithm for the DSD reaction network. The condensation algorithm for CRN uses slow reactions. The approximate rate model for DNA domain level systems. Last, they performed multiple case studies that compared their model with real experiments. Peppercorn analysis examines the DSD reactions at the domain level, which can be rigorously analyzed without knowing the specific type of nucleic acid or polymer.

## 5 Summary

DNA computing is a promising technology that combines DNA nanotechnology and computer science. It exploits the massively parallel nature of molecules. DSD is the simplest DNA computing strategy and involves the inputs and outputs of signal DNA strands. What’s more, DSD can be programmed and cascade into complex chemical reaction networks. DSD integrated circuits have been implemented to realize machine learning and artificial neural networks. With the help of molecules and biotechnologies, the DSD computational system can be used in various applications. DSD with CRISPR technology has been applied to construct intracellular circuits and biosensing. DSD had been demonstrated its ability to detect cancer cells. DSD is also useful in data storage and encryption. Although DSD is already widely applied, it has a lot of development potential. DSD is a simple tool, and its development depends on the cross-fertilization of other biotechnologies.
